# Obituary

**Published:** 1856-10

**Authors:** 


"DIED. In this city, on Thursday morning, the 10th inst., at 8¼ o'clock, after a protracted illness, Prof. John Locke, in the 65th year of his age."

Soon after the last issue of the *Register*, the above appeared in our city papers. We leave others to write eulogies. They are well for those who can. Our pen utterly refuses. Whether we view him as a man—a philosopher—a preceptor—a *friend*—all we can write is—

Dr. JOHN LOCKE.

"*His monument shall be his name alone.*"

